# Investigating the temperature distribution behavior and flow parameters of argon fluid in a nanochannel with changing dimensions of the obstacle using the molecular dynamics (MD) method

**DOI:** 10.1016/j.heliyon.2024.e24065

**Published:** 2024-01-06

**Authors:** Omid Ali Akbari, Ebrahim Shirani, Mohsen Saghafian

**Affiliations:** a Mechanical Engineering Group, Pardis College, Isfahan University of Technology, Isfahan, 84156-83111, Iran; b Department of Mechanical Engineering, Isfahan University of Technology, Isfahan, 84156-83111, Iran

**Keywords:** Nanochannel, LAMMPS software, Rectangular obstacles, Density distribution, Heat flux, Molecular dynamics

## Abstract

This article, examines the flow of argon inside a nanochannel with respect to the molecular dynamics (MD) in the free molecular flow regime using LAMMPS software. The nanochannel is made of copper featuring a square cross-section and obstacles of varying dimensions and values. In this study, the flow of argon fluid is three-dimensional. To gain a deeper understanding of the effect of solid walls within the nanochannel and their influence on flow behavior, the research is simulated in a nanochannel with all side walls for the 3D model and without side walls for the 2D model. This research assesses the effect of the obstacles’ dimensions and values on the nanochannel wall surface and areas above the wall surface. The total dimensions of all simulated two- and three-dimensional atomic structures with a square cross-section are assumed to be 60 × 60 × 100 Å^3^. and the presence of square obstacles (with dimensions of 8 × 8 × 8 Å^3^) and rectangular obstacles (with dimensions of 8 × 18 × 8 Å^3^) is examined. This study seeks to understand the influence on flow behavior, temperature distribution, density, heat flux, velocity, and thermal conductivity coefficient. This study is simulated using a time step of 1 fs for 10,000 time steps, involving approximately 10,000–15,000 argon and copper atoms. The results of this research indicate that obstacles with structures of P and R and larger dimensions increase the number of solid atoms exhibiting stronger attractive forces. Compared to a smooth nanochannel, the thermal exchange between fluid and solid atoms results in a density increase of 17.5 % and 17.3 %, respectively. On the other hand, in the 3D nanochannel, the sidewalls of the nanochannel have reduced the effect of the presence of R and P obstacles with larger dimensions, which comparing to a smooth nanochannel, have increased the density by 8.21 % and 7.53 %, respectively. The obstacles with different spatial positions (P and R structures) in the two-dimensional nanochannel cause a rise in the thermal conductivity coefficient. The P structure obstacles have a better effect on the thermal conductivity coefficient in the 2D nanochannel compared to the R structure. In the three-dimensional nanochannel, utilizing smaller obstacles proves to be more effective because it results in better atom distribution or temperature distribution due to increased atomic collisions in the central region compared to the wall regions.

## 1 Introduction

Nano-dimensioned fluid flows, referred to as nanoflows, significantly influence the characteristics of numerous engineering and biological systems. One application of these flows is ion channels, which inherently lead to the formation of nanotubes within the membranes of all biological cells [1]. The DNA movement inside a nanopore is a great example for the nanoflow's applications. Other applications of nanoflows include fuel cell systems, drug delivery systems, chemical and biological measurement devices, and any system where nanoflows are essential and necessitate precise and thorough analysis [2,3]. In recent years, the investigation of nanoflows has gained significant importance and interest among researchers. Hens et al. [4] conducted molecular dynamics simulations of liquid argon to examine the bubble formation mechanism on a platinum substrate influenced by a specific surface structure known as Ramord. The findings of this study revealed that bubble formation on a non-wetting surface is not easily achieved. Additionally, hydrophilic surfaces create favorable conditions for bubble nuclei and the development of vapor films. Noorian et al. [5] investigated the flow behavior of argon in its liquid phase in a smooth nanochannel in the presence of roughness via MD simulations. Their findings indicated that the presence of walls absorbs the energy of the flow and increases the density layer, especially in the vicinity of the wall. This effect becomes more pronounced with increasing roughness height, leading to the formation of a secondary layer phenomenon. The researchers demonstrated that the height and shape of the roughness play a crucial role in fluid flow, and increasing the roughness ratio results in reduced speed and increased viscosity oscillation near the wall. In another study, Noorian et al. [6] used MD simulations to assess the effects of nanochannel roughness with cubic and spherical shapes of varying heights. The fluid under investigation in this research was liquid argon. The researchers presented the results of the impact of the aforementioned roughness on velocity and density profiles, demonstrating that with increasing roughness height, both density and velocity decrease. This is because roughness directly affects intermolecular forces, increasing repulsion and reducing the effect of attractive forces between adjacent atoms in the area influenced by roughness elements. Pengfei et al. [7] utilized MD simulations to investigate the electromagnetic flow inside a rough nanochannel. In this study, the fluid consisted of water molecules, and two types of rectangular teeth were utilized in each simulation, which were arranged alternately and regularly on the nanochannel surface. The researchers' results demonstrated that the presence of roughness reduces the concentration of water molecule oscillation near the solid wall. Additionally, this oscillation is further reduced as the roughness height increases and the distance between the teeth decreases. Cao et al. [8] utilized MD simulations to examine the effects of rectangular, triangular, sinusoidal, and irregular wave-shaped roughness inside a platinum channel with a height of 0.1 μm and argon gas flow. They concluded that the boundary conditions of slip and non-slip are influenced by surface roughness and the Knudsen number. This is because the friction coefficient of argon gas flow in a microchannel with a rough surface is higher than that of a smooth surface. Furthermore, the friction coefficient increases with a decreasing Knudsen number and increasing surface roughness. The shape of the roughness also plays a significant role in determining boundary conditions and friction properties. Sofos et al. [9] studied the argon flow in its liquid phase through a krypton nanochannel via MD simulations. The nanochannel examined in this research had smooth and rough surfaces, respectively. In the rough state, only the upper surface was considered rough, and the lower surface of the nanochannel was smooth in all cases. Also, the roughnesses were considered in three different ways in terms of width and distance between them. These researchers reported that the shear stress is higher on the rough surface than the smooth one, which leads to the change of the velocity values and the non-linearity of its profile, making the shape of the velocity profile tend to be exponential. They found that the stress levels on the tooth layers were higher than in the spaces between the teeth, resulting in higher velocity profiles in these areas. Additionally, the rough surface had a higher viscosity value compared to the smooth surface. Galea et al. [10] used molecular dynamics simulation to investigate the effects of solid roughness on slip boundary conditions during the shear flow of fluid and reported that the number of atoms in fluid layers cannot remain constant with changes in surface structure. They also reported the range of changes in the density oscillation amplitude with changes in surface smoothness and concluded that when thermodynamic parameters are constant, the surface structure affects the fluid viscosity value. Tang et al. [11] MD simulations to investigate the heat transfer and flow behavior of argon in the liquid phase between two parallel plates at the nanoscale while simultaneously controlling motion and temperature field solution. In this research, the temperature of the upper copper plate is higher than the lower plate and is moving under the influence of external force. In this research, the displacement heat transfer coefficient can be calculated according to the amount of heat flux exchanged between the fluid and the plates. They found that the heat transfer coefficient between liquid argon flow and the copper plate was lower than predicted by other studies but still within a similar order of magnitude. Chen and Zhang [12] studied the molecular dynamics of fractal rough nanochannels with varying degrees of roughness. They used fractal surface properties to calculate thermal conductivity on rough surfaces and determine surface topography's impact on thermal conductivity at the liquid-solid interface. They analyzed the effect of surface roughness on temperature profile, liquid atomic paths, interfacial interaction energy, and surface conductivity. Their findings revealed that a thermal jump at the solid-liquid interface occurred regardless of surface conditions and that surface roughness reduced the surface temperature jump. In a study by Mukherjee et al. [13], molecular dynamics simulations were used to simulate the transition of water from liquid to gas phase on a silicon nanochannel with grooves. The simulation was conducted under the influence of constant heat rate and flux. The study revealed that the fluid is primarily confined to the grooves at low temperatures. However, as the heat flux increases, the molecules vibrate more vigorously, and the fluid spreads to other regions. Also, the gradual dilution of molecules leads to the formation of vapor bubbles, which increase in size over time, and the growth rate of these bubbles is a function of surface geometry parameters such as crack height, crack width, crack type, and crack distance.

Researches on MD of fluid movement through non-smooth nanochannels with various geometry configurations [[14], [15], [16]] reveals that the flow patterns in distinct areas of the nanochannel have not been addressed. In earlier research carried out by the authors of this study [17], they discussed the existence of obstacles in a copper nanochannel with varying roughness values. The investigation focused on the flow behavior, temperature distribution features, and flow parameters. Furthermore, the impact of obstacles and their spatial positioning in various areas of the nanochannel has not been examined separately and concurrently, and the influence of these factors on flow parameters and other nanochannel regions, particularly near-wall and mid-flow sections, has not been distinctly explored in any research. Furthermore, the impact of obstacles and their spatial positioning in various areas of the nanochannel on flow parameters has not been examined separately and concurrently. In addition, the influence of these factors on different regions of the nanochannel, specifically, in the vicinity of the wall and mid-flow sections, has not been distinctly explored in any research. The present study investigates the effect of surface factors (R structure) and cross-sectional (P structure) with varying values in two- and three-dimensional square nanochannels under the influence of external force. Altering the flow parameters are extensively investigated using hybrid Lennard-Jones potential function (EAM).

## 2 Problem statement

**Figure (1):**
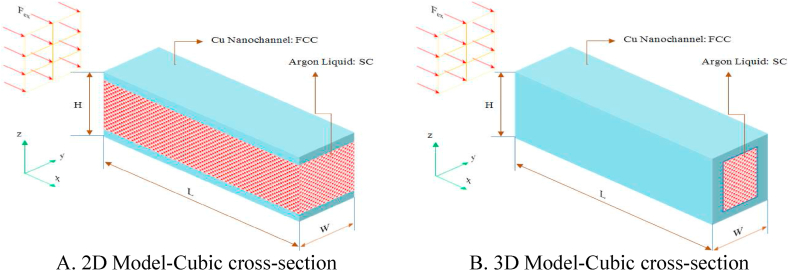
Schematic of the simulated model of the cross-section of copper nanochannels under investigation.

In the present paper, the argon's flow is simulated in a smooth nanochannel with 2D and 3D square obstacles to determine the density, temperature, velocity, heat flux, and thermal conductivity coefficient profiles of fluid atoms. Fig. 1(A, B) presents a schematic of the sample nanochannels in smooth and obstacle-free conditions, without surface or cross-sectional obstacles, in two- and three-dimensional states.

Table (1), summarizes the value of some of the parameters and dimensions assumed for this problem.

**Table (1):** Value of some of the parameters and dimensions.

Parameter	What it represents	Value
L	Nanochannel length	100 Å
H	Channel height	60 Å
W	Channel width	60 Å
t	Walls' thickness	5 Å
D_h_ = 4A/P	Hydraulic diameter	50 Å
*Kn* = λ/D_h_	Knudsen number	10.8
A = (H-2t)(W-2t)	Cross-sectional area	2500 Å^2^
P = 2((H-2t)+(W-2t))	Wetted perimeter of the nanochannel cross-section	200 Å

**Figure (2):**
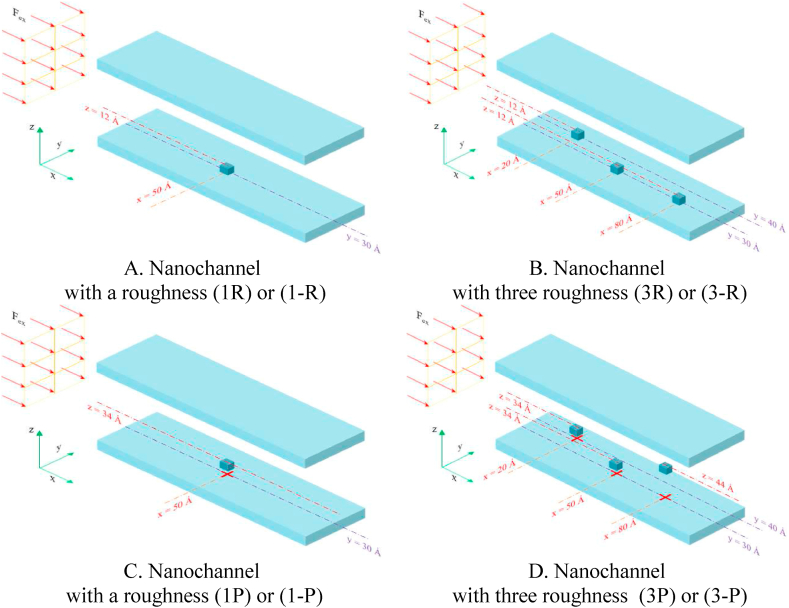
Schematic of the simulated square nanochannel with square obstacles of P and R structures with dimensions (8 × 8 × 8 Å^3^) and different values.

**Figure (3):**
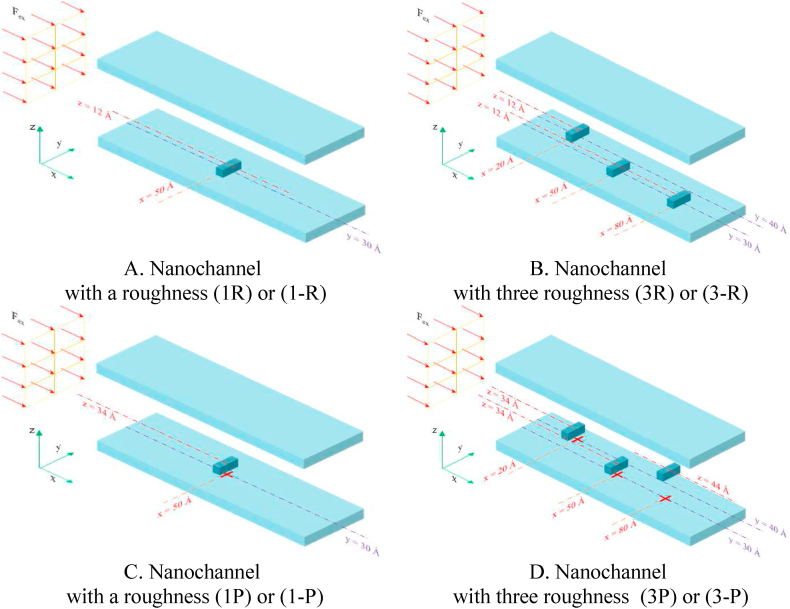
Schematic of the simulated square nanochannel with rectangular obstacles of P and R structures with dimensions (8 × 8 × 8 Å3) and different values.

In Fig. 1(A, B), every wall comprises three copper atoms layers. They are considered as face-centered cubic lattice and the lattice constant (*a*) is 3.61 Å. At the start of the simulation the liquid argon atoms have a simple cubic structure and their *a* is 3.4 Å. Fig. 2(A–D) and 3(A - D) depict square-section nanochannels featuring obstacles with P and R structures of varying dimensions and values. These nanochannels are considered in both 2D (2 walls) and 3D (4 walls) configurations. The total dimensions of all simulated two- and three-dimensional atomic structures with a square cross-section are assumed to be 60 × 60 × 100 Å^3^. and the presence of square obstacles (with dimensions of 8 × 8 × 8 Å^3^) and rectangular obstacles (with dimensions of 8 × 18 × 8 Å^3^) is examined. In this simulation, the impact of increasing the number of obstacles and altering the dimensions of obstacles (specifically, their width) on flow parameters and temperature field is examined, with initial conditions set at a temperature of 300 K in these structures. The atomic samples are subjected to a canonical ensemble (NVT) for 10 ns. Following this, the particles of the fluid undergo an externally induced force with a magnitude of 0.1 eV/Å to facilitate fluid's movement within the nanochannel. The initial conditions in these structures involve a wall temperature of 300 K for all cases and atomic samples for 10 pico-seconds (ps) with a 1 fs time step to guarantee complete system equilibrium.

## 3 Governing equations

In a molecular dynamics simulation, the primary objective is to determine the phase space by employing numerical techniques to solve Newton's equations through Hamilton's equations. Physical properties depend on the position and momentum of each particle (N particle). As such, a general physical quantity (A) at a specific time (t) can be defined as illustrated in equation (1) [18].

(1) $$A\left(t\right)=f\left({\vec{r}}_{1}\left(t\right),{\vec{r}}_{2}\left(t\right),...,{\vec{r}}_{N}\left(t\right),{\vec{p}}_{1}\left(t\right),{\vec{p}}_{2}\left(t\right),...,{\vec{p}}_{N}\left(t\right)\right)=A\left({\vec{r}}^{N}\left(t\right),{\vec{p}}^{N}\left(t\right)\right)$$

Additionally, its mean value is derived via equation (2) [18].

(2) $${\left\langle A\right\rangle }_{time}=\frac{1}{M}\sum_{i=1}^{M}A_{i}\left(t\right)$$

where, (M), is the total time steps and (i) is the simulation time steps. Conversely, macroscopic characteristics consistently correspond to the collective average of a statistical ensemble, signifying an average attribute for molecular systems and are provided via equation (3) [18]:

(3) $$A_{obs}={\left\langle A\right\rangle }_{ens}$$

where (A) is any property that can be quantified. The Newton's equation (Equation (4)) determines the particles' positions (${\vec{r}}^{N}$) in the MD simulation.

(4) $${\vec{F}}_{i}\left(t\right)=-\frac{\partial U\left({\vec{r}}^{N}\right)}{\partial {\vec{r}}_{i}}=m{\ddot{\vec{r}}}_{i}\left(t\right)=\sum_{i}\sum_{i\neq j}{\vec{F}}_{ij}+\sum_{i}\sum_{i\neq j}{\vec{F}}_{ij,w}+{\vec{F}}_{ext}$$

Here, (${\vec{F}}_{i}\left(t\right)$) is the total force applied on the particle (i) from different sources. The term ($\sum_{i}\sum_{i\neq j}{\vec{F}}_{ij}$) is the force applied from other particles. The term ($\sum_{i}\sum_{i\neq j}{\vec{F}}_{ij,w}$) is the force applied from solid walls. The term (${\vec{F}}_{ext}$) is the external force and creates the non-equilibrium conditions during non-equilibrium MD simulations. Afterwards, the system's reaction to the (${\vec{F}}_{ext}$) determines the particles' positions. If we integrate Equation (4) two times, the particle's velocity and position is reached. The trajectory of particles can be reached if the integration is repeated for hundreds of time intervals. Particle trajectories can be used to determine the macroscopic properties. Any thermodynamic property ($A\left({\vec{r}}^{N}\left(t\right),{\vec{p}}^{N}\left(t\right)\right)$) can be measured via the position and momentum of every particles in the system. As time advances, various values of thermodynamic property will alter accordingly. Eventually, each macroscopic property measured experimentally (A) would be the mean value of ($A\left({\vec{r}}^{N}\left(t\right),{\vec{p}}^{N}\left(t\right)\right)$) during a long time period, which is calculated according to equation (5) [19].

(5) $$A_{obs}={\left\langle A\right\rangle }_{time}={\left\langle A\left({\vec{r}}^{N}\left(t\right),{\vec{p}}^{N}\left(t\right)\right)\right\rangle }_{time}=\lim_{t\to \infty }\frac{1}{t}\int_{t_{0}}^{t_{0}+t}A\left({\vec{r}}^{N}\left(\tau \right),{\vec{p}}^{N}\left(\tau \right)\right)d\tau$$

Here, ($A_{obs}$) is the value of a specific property reached from the experimental data. The term ($A\left({\vec{r}}^{N}\left(\tau \right),{\vec{p}}^{N}\left(\tau \right)\right)$), can be obtained from the position and momentum of the system's particle, (t) is the entire time during which calculating the term (A) was done. (${\left\langle A\right\rangle }_{time}$) is the time average of (A). For equilibrium thermodynamic systems, the time average (${\left\langle A\right\rangle }_{time}$) is nearly equivalent to the ensemble average. The ensemble average is obtained from equation (6) [20]:

(6) $$A_{m}=\frac{1}{t}\int_{t_{0}}^{t_{0}+t}A\left({\vec{r}}^{N}\left(\tau \right),{\vec{p}}^{N}\left(\tau \right)\right)d\tau$$

where ($t_{0}$) represents the initial time, ($A_{m}$) denotes the average property value, and (t) signifies the measurement time. Consequently, for thermodynamic systems in equilibrium, equation (7) is valid:

(7) $${\left\langle A\right\rangle }_{time}=A_{m}$$

By employing Equation (6), the kinetic energy and temperature can be determined according to the phase trajectory from equation (8) [18].

(8) $$\left\langle E_{k}\right\rangle =\lim_{t\to \infty }\frac{1}{t}\int_{t_{0}}^{t_{0}+t}E\left({\vec{p}}^{N}\right)dt=\frac{3}{2}Nk_{B}T$$

Here, (T) is temperature, ($k_{B}$) is the Boltzmann constant, (N) is the system's total molecules, (${\vec{p}}^{N}$) is the particles' momentum, and ($\left\langle E_{k}\right\rangle$) is the system's average kinetic energy. In equation (9), to assess the pressure, we should consider two terms. ($P_{m}$) is associated with the momentum transferred by atoms crossing a hypothetical plane over a (dt) time interval. ($P_{f}$) is obtained from the momentum force of the particles on the other sides of the plane [18].

(9) $$PV=Nk_{B}T+\left\langle W\right\rangle \;\to \;P=P_{m}+P_{f}=\frac{2N}{3V}\left\langle E_{k}\right\rangle +\frac{1}{3V}\left\langle \sum_{i=1}^{N}\sum_{i<j}^{N}\vec{F_{ij}}.\vec{r_{ij}}\right\rangle$$

Here, (V) is cell volume, (${\vec{F}}_{ij}$) is the force atom (j) applies on atom (i), where (${\vec{r}}_{ij}$) represents the distance from atom (j) to (i). We can obtain the virial using the vector production of particle coordinates and the force exerted on them $\left[W=-\frac{1}{3}\left\langle \sum_{i=1}^{N}\sum_{i<j}^{N}\vec{F_{ij}}.\vec{r_{ij}}\right\rangle \right]$. Finally, the total energy can be calculated via equation (10):

(10) $$E=E_{k}\left({\vec{p}}^{N}\right)+\sum_{i=1}^{N}\sum_{i<j}^{N}U\left({\vec{r}}_{ij}\right)$$

In Equation (10), ($U\left({\vec{r}}_{ij}\right)$) is the potential energy between atom i and j. The Lennard-Jones equations (6)–(12) is introduced as equation (11) [21]:

(11) $$r_{ij}\leq r_{c}\;U\left(r_{ij}\right)=4\varepsilon \left[{\left(\frac{\sigma }{r_{ij}}\right)}^{12}-{\left(\frac{\sigma }{r_{ij}}\right)}^{6}\right]$$

Here, (σ) is the particle's diameter, and is equal to the problem's length scale. ($r_{ij}$) is the distance between the atom i and j where the potential function equals zero. (ε) is the interaction strength between particles. For modeling the fluid-solid interaction, the generalized form of the Lennard-Jones potential function is used, and instead of (σ) and (ε) from, ($\sigma_{\text{sf}}$) and ($\varepsilon_{\text{sf}}$) are employed. Equation (12) presents the general form of this function [21]:

(12) $$U_{wall}\left(r\right)={4a\varepsilon }_{sf}\left\{\left[{\left(\frac{\sigma_{sf}}{r}\right)}^{12}-\beta {\left(\frac{\sigma_{sf}}{r}\right)}^{6}\right]\right\}$$

In relation (12), (r) is the distance between the fluid particles and the solid wall. α is the potential energy coefficient, which indicates the hydrophilicity of the surface, while (β) is also a potential energy coefficient that determines the hydrophobicity of the surface [22,23].

## 4 Geometry, solution method and assumptions

### 4.1 Geometry of the problem

This study introduces geometries associated with the MD flow simulation of fluid argon utilizing LAMMPS software within a square nanochannel (dimensions 60 × 60 × 100 Å^3^) featuring square and rectangular obstacles of varying sizes. The obstacles have equal dimensions, with the square ones measuring 8 × 8 × 8 Å^3^ and the rectangular ones 8 × 18 × 8 Å^3^. The simulation examines the impact of increasing obstacle dimensions and quantity on flow parameters and temperature fields, considering the initial conditions. A significant parameter involves the aspect ratio of the inner wall's geometric irregularities. Exploring the geometric shape of the inner wall's irregularities (R structures) and the cross-sectional obstruction factors (P structures) on the Poiseuille Flow of argon fluid within a copper nanochannel represents one of the innovative aspects of this study.

### 4.2 Assumptions and boundary conditions

Using the molecular dynamics method, specific general rules have been considered in investigating the geometric shape of the inner wall irregularities on argon flow in a copper nanochannel. In this investigation the atomic mass of copper is considered 63.55 g/mol and that of argon is considered 39.95 g/mol. The hybrid Embedded Atom Method (EAM) potential function is used to study the atoms' interactions inside the nanochannel. The EAM potential function constant coefficients are considered in Table (2).

**Table (2):** EAM potential function constant coefficients.

$r_{ij}$	3.4 Å
$\varepsilon_{Ar-Ar}$	0.0104 eV
$\sigma_{Ar-Ar}$	3.4 Å
$r_{ij}$	3.872 Å
$\varepsilon_{Ar-Cu}$	0.0653 eV
$\sigma_{Ar-Cu}$	3.4 Å

The constant coefficients available in EAM Metal EAM file [24] was used for the copper atom interactions. The walls of the nanochannel are designated as rigid and immovable. In all analyses, fluid particle movement is considered in the X direction. Fixed boundary conditions are considered for the Y and Z directions. While along the X-axis, periodic boundary conditions is considered to remove the system's surface effects. The system's number of particles remains constant since when a particle enters the system form one side, another particle exits from the other. The primary wall temperature for all cases is set at 300 K. To make sure the system remains in equilibrium, the time steps are considered to be 0.1 fs, and the atomic samples are assessed for 100 ns with a canonical ensemble (NVT). An external force equal to (0.1 eV/Å) is exerted on the fluid particles, and the problem under non-equilibrium conditions is studied for 10 ps with the same time being 1 fs. In order to ease the fluid's movement along the channel, we divide different regions of the nanochannel into 38 bins along the transverse Y direction. Table (3) provides the number of atoms that are studied in different cases and states.

**Table (3):** Number of atoms investigated in the simulation cases with a square shape nanochannel.

Cross-section of the nanochannel	Number of R and P	R and P dimensions	Number of argon atoms	Number of Cu atoms	Total number of atoms
2D	–	–	4320	5712	10032
3D	–	–	4320	10416	14736
2D	N = 3P	P: (8 × 8 × 8 Å^3^)	4239	5834	10073
3D	N = 3P	P: (8 × 8 × 8 Å^3^)	4239	10538	14777
2D	N = 3R	R: (8 × 8 × 8 Å^3^)	4293	5778	10071
3D	N = 3R	R: (8 × 8 × 8 Å^3^)	4293	10482	14775
2D	N = 3P	P: (8 × 18 × 8 Å^3^)	4158	5992	10150
3D	N = 3P	P: (8 × 18 × 8 Å^3^)	4158	10696	14854
2D	N = 3R	R: (8 × 18 × 8 Å^3^)	4266	5878	10144
3D	N = 3R	R: (8 × 18 × 8 Å^3^)	4266	10582	14848

## 5 Results and discussion

In this section of the study, the discussed parameters pertain to the simulation of argon fluid flow within two- and three-dimensional square nanochannels (measuring 60 × 60 × 100 Å^3^) containing square and rectangular obstacles with varying quantities and dimensions of 8 × 8 × 8 Å^3^ and 8 × 18 × 8 Å^3^. In these graphs, the P index denotes the structure of obstacles or obstructions situated above the nanochannel wall surface. In contrast, the R index represents the obstacles' structure on the nanochannel surface. The R–P index is based on the concurrent presence of both obstacle structures within the nanochannel.

### 5.1 Validation

**Figure (4):**
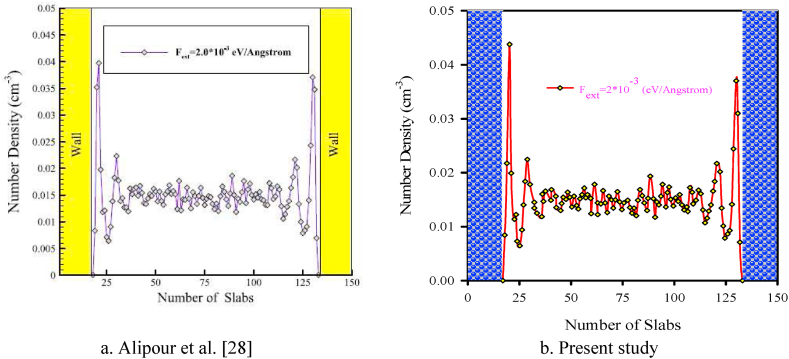
Density behavior across the rough nanochannel.

**Figure (5):**
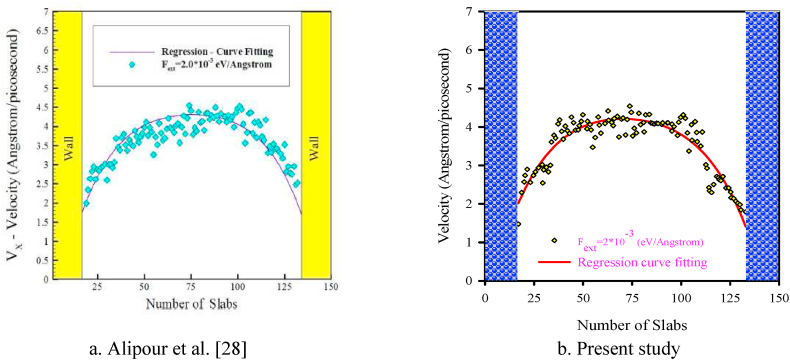
Axial velocity profile diagrams of argon flow in a smooth nanochannel along the width direction.

The validation results of this study is demonstrated in Fig. 4(a and b) and 5(a, b) where the results are compared to those of Alipour et al. [25] in a 3D nanochannel made of platinum encompassing argon fluid for parallel flow on which an external force F_ext_ = 0.002 eV/Å was applied. The changes in the density and velocity of argon atoms is assessed in the 3D nanochannel with rectangular roughness. In Alipour et al. [25] study, the heat transfer and MD behavior of argon flow was investigated in a 3D rectangular nanochannel with various wall surface roughnesses via a Hybrid EAM LJ potential function. They studied the equilibrium phase by setting an initial temperature of 300 K. The external force was applied to study the time-dependent simulations of parallel argon gas flow to assess the velocity and density fields within the channel. In another research by Alipour et al.'s [28], to assess the flow properties, the atomic binning was done in transverse simulation. They divided different regions of the nanochannel into 150 bins along the transverse Y direction. Fig. 4(a and b) and 5(a, b) demonstrate the acceptable agreement of the present study with those of Alipour et al. [25].

### 5.2 Total and potential energy of the simulated systems at the time step

The system's total energy (kinetic energy + potential energy) is constant over time in any isolated system (NVE microcanonical system) and here is equal to Hamiltonian H when selecting proper symbols (${\vec{r}}^{N}=\left\{{\vec{r}}_{1},{\vec{r}}_{2},...,{\vec{r}}_{N}\right\}$) and (${\vec{p}}^{N}=\left\{{\vec{p}}_{1},{\vec{p}}_{2},...,{\vec{p}}_{N}\right\}$). In a system containing N molecules, the Hamilton H function (Equation (13)) is the sum of kinetic energy and potential energy of each molecule with (i) index with a coordinate (${\vec{r}}_{i}$) and a momentum (${\vec{p}}_{i}$) and is derived as:

(13) $$H\left({\vec{r}}^{N},{\vec{p}}^{N}\right)=K\left({\vec{p}}^{N}\right)+U\left({\vec{r}}^{N}\right)=\frac{1}{2m}\sum_{i}{\vec{p}}_{i}^{2}+U\left({\vec{r}}^{N}\right)=E$$

To solve this function, ${\vec{f}}_{i}$ (the effective force on the molecule i) and ${\vec{\tau }}_{i}$ (the torque on the molecule i) should be assessed via the potential energy (U). This function determines the equilibrium distribution function of molecular positions and momenta as well.

Differentiating equation (13) will result in Hamiltonian motion equations (equation (14) and (15)).

(14) $$\frac{\partial H}{\partial {\vec{p}}_{i}}=\frac{{\vec{p}}_{i}}{m}={\dot{\vec{r}}}_{i}$$

(15) $$\frac{\partial H}{\partial {\vec{r}}_{i}}=\frac{\partial U}{\partial {\vec{r}}_{i}}=-{\dot{\vec{p}}}_{i}$$

**Fig. 6:**
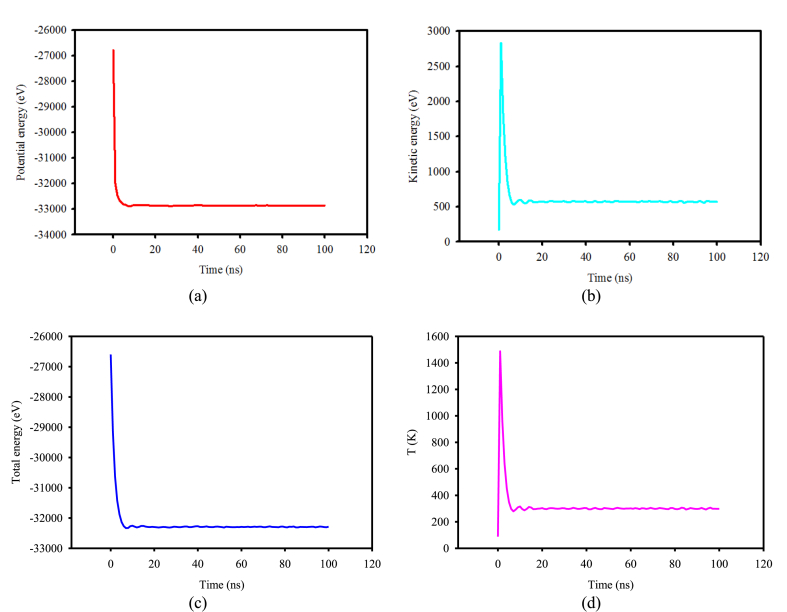
Graphs of temperature field, potential energy, kinetic energy, and total energy variations for an ideal three-dimensional copper nanochannel over 100 ns.

Fig. 6(a–d) shows the potential energy, kinetic energy, total energy, and temperature of the particles present in the three-dimensional nanochannel containing argon fluid as a function of simulation time. The system's total energy is the sum of the kinetic and potential energies of the atoms. The potential energy depends on the type of interatomic interaction, while the kinetic energy is a function of the external force applied to the system. Based on the results obtained from the simulations performed for the modeled structure, the minimum total and kinetic energy values are approximately −32255.868 eV and 601.29 eV, respectively, which occur after about 10 ns. The potential energy value in this time interval is approximately −32857.161 eV. Incorporating solid walls into the copper nanochannel results in more copper atoms within the simulation, raising the structure's total potential energy. The system attains stability after around 10 ns, and after 100 ns, the total energy, potential energy, and kinetic energy stabilize at approximately −32287.091 eV, −32854.244 eV, and 567.15 eV, respectively.

For the temperature field variations of the fluid and nanochannel, reaching 300 K is possible after 10 ns and with minor changes. This behavior is due to added solid walls and more contact between the fluid and the solid walls’ atoms (extra atoms comparing to the 2D state). Ultimately, it remains stable at this temperature after 100 ns have passed.

### 5.3 Investigating density variations

**Figure (7):**
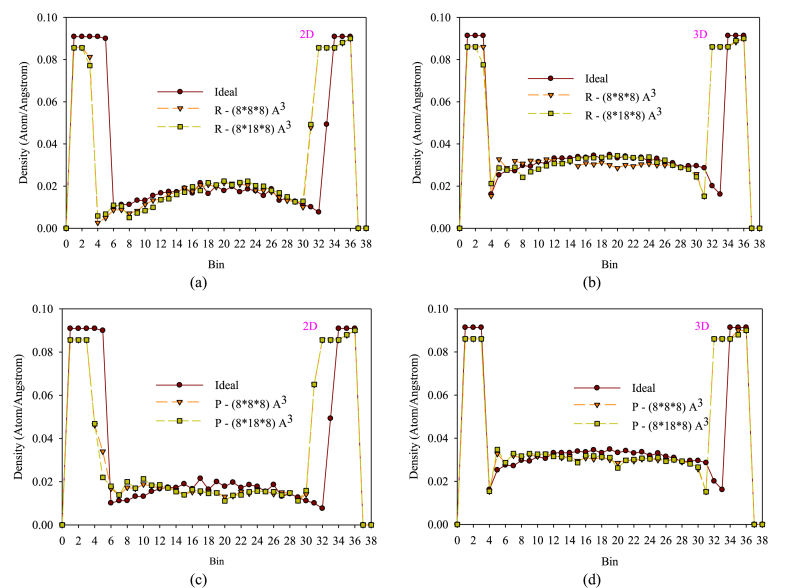
Diagrams of fluid atom density behavior across the nanochannel with changes in obstacle dimensions with structures (P) and (R).

Fig. 7(a–d) depicts the density profile patterns of fluid atoms in the nanochannel's width when the dimensions of obstacles featuring structures (P) and (R) expand to a value of 1, compared to a smooth nanochannel in both two and three dimensions. The provided illustrations depict the impact of fluid atom buildup in various areas of the nanochannel by illustrating density patterns. In both two and three-dimensional nanochannels across all examined scenarios, the strong atomic interaction between argon and copper atoms in solid wall areas, combined with a reduction in kinetic energy of fluid atoms due to collisions with wall atoms and a decrease in fluid atom temperature, result in an increased atomic accumulation in these regions, which in turn leads to a higher density. In these areas, the presence of obstacles with structure R and the reduced flow velocity caused by fluid-solid atom collisions contribute to the enhancement of fluid atom density as obstacle dimensions increase, particularly in regions near the wall. In wall regions, a larger obstacle size leads to a greater amount of solid surface with lower temperatures or collision surfaces. Increasing solid surfaces or heat transfer surfaces can result in higher thermal wear. Conversely, expanding the obstacles leads to greater blockage within the nanochannel, ultimately raising the density and prolonging the fluid atom stoppage in solid surface areas. Beyond the wall regions, as we progress towards the nanochannel's center (the bulk flow region), there is a notable decline in the density diagrams. The increased copper atoms number in the walls in the three-dimensional nanochannel contributes to a more significant density rise in the central or bulk flow section along the nanochannel's height, owing to greater thermal exchange. As a result, the level of the density diagrams for the three-dimensional nanochannel increases compared to those in the two-dimensional case. Due to their varying heights, the inclusion of P structure obstacles affects not only the central flow regions but also the sub-surface or near-wall sections by causing an increase in density. The motion of fluid atoms around these obstacles, along with direct collisions with them, leads to the disruption of the direct flow movement due to the obstruction of the flow cross-section in these regions. This results in lateral collisions of fluid atoms with wall atoms, consequently increasing density. The density change diagrams' behavior also indicates that the heterogeneity of these diagrams is quite pronounced with the R structure obstacles.

The highest density increase is related to the presence of roughness (R) and obstacles (P) with larger dimensions in the two-dimensional nanochannel. The reason for this increase is due to the presence of two upper and lower walls, which, due to the presence of roughness and obstacles, the number of solid atoms with strong attraction force increases, and the temperature exchange of fluid with solid atoms leads to an increase in density by 17.5 and 17.3, respectively. On the other hand, in the three-dimensional nanochannel, the effect of the presence of the side walls of the nanochannel has caused a lower effect of the presence of roughness and larger barriers (P) by 8.21 and 7.53, respectively, compared to the smooth nanochannel on the increase in density. It seems that the effect of the presence of obstacles (P) has increased the fluid density in the 3D nanochannel due to affecting the areas further away from the solid surface. In general, the density behavior in two and three-dimensional nanochannels is affected by the concentration of fluid atoms in different areas, and the presence of roughness and obstacles with larger or smaller dimensions can affect the atomic distribution.

**Figure (8):**
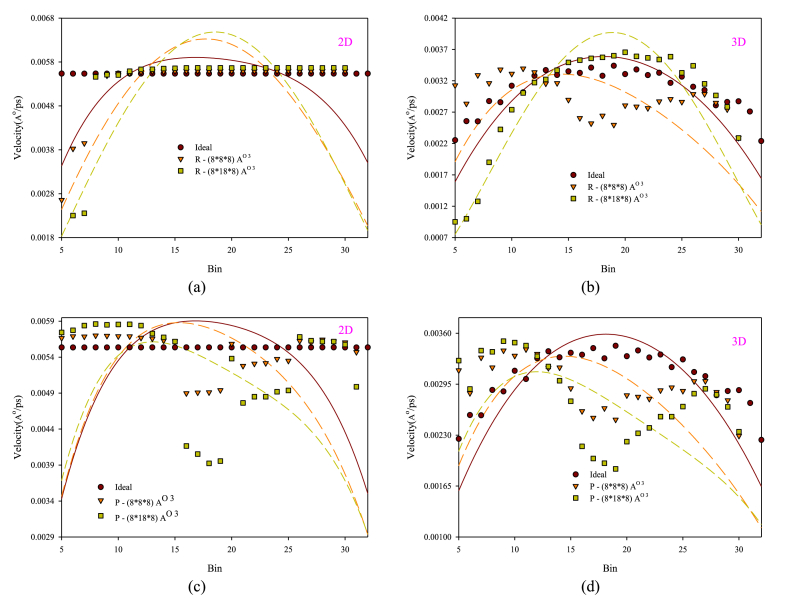
Changes in axial flow velocity profile with the presence of obstacles with different dimensions and structures.

Fig. 8(a–d) shows the behavior of axial flow velocity variations in the transverse direction of the nanochannel with the presence of different structures and dimensions of obstacles. In these charts, a 4th-order non-linear curve fitting is performed for each case with the same color display, and it is compared with the results of the smooth nanochannel. The motion of fluid atoms in the nanochannel is influenced by the application of external forces. Due to the collision between fluid atoms and solid wall atoms, the presence of solid walls can also affect the behavior of velocity profiles. On the one hand, obstacles obstruct the flow cross-section, impeding and altering the fluid atoms' path as they move through the nanochannel. The existence of wall atoms and obstacles causes the velocity profile to deviate from its optimal form. In each graph, enlarging the obstacles leads to an irregular velocity profile. The introduction of R-structured obstacles on the solid wall decreases the wall atoms' velocity in that region and disturbs the fluid's linear flow movement. This behavior leads to localized blockage of the flow cross-section in the nanochannel and increases the extremum of the velocity profile in both 2D and 3D nanochannels. This behavior occurs with a higher intensity for two-dimensional nanochannels than three-dimensional ones as the size of the obstacles increases. The P structure obstacles in areas higher than the flow level cause a stagnation in the flow velocity in central or bulk flow regions. Consequently, the velocity profiles at the centerline of the flow, especially with increasing dimensions of obstacles with a P structure, experience a higher velocity stagnation, and the flow velocity compared to a smooth nanochannel experiences higher stagnation and flatter velocity profile peaks. Moreover, in the velocity profiles, the presence of larger obstacles causes a deviation in the velocity profile and the formation location of the maximum speed, particularly for the three-dimensional flow case, which is more noticeable, and the flow profile deviation toward the lower wall is more significant. This observed behavior appears to be because of the of R-arranged obstacles on the lower wall and P-arranged obstacles near the lower wall, intensifying the fluid atom velocity reduction in these areas.

Conversely, the illustrations in Fig. 8(a–d) depict the axial velocity profiles incorporating slip effects on solid surfaces, featuring obstacles with a value of 1 and varying dimensions comparing to a smooth nanochannel. The slip velocity correlates with both the cross-sectional velocity gradient and the Knudsen number, however, the slip length has a direct relationship with shear stress. As the continuity assumption and no-slip boundary condition do not hold true at the nanoscale, atomic interactions between solid surfaces and fluid atoms become entirely random. This results in varying velocities for each of the atoms on the solid wall, which do not align uniformly with the wall's velocity. Consequently, the motion of fluid atoms along the solid surface, besides being influenced by external forces, may exhibit distinct behaviors due to the presence of obstacles of varying dimensions. The slip velocity behavior on the upper and lower walls differs, leading to asymmetry in the velocity diagrams. This asymmetry arises from alterations in surface and cross-sectional properties caused by the R-arranged and P-arranged obstacles. These variations also impact the peak of the velocity profile, resulting in notable changes. Conversely, altering the dimensions of obstacles modifies the behavior of axial flow velocity profiles compared to those in a smooth nanochannel, potentially impacting the fluid atoms' slip length and slip velocity on the copper wall. Enhancing the obstacles to the walls and central regions of the nanochannel results in an increased in the average and maximum value of the velocity due to the reduction in the nanochannel's cross-sectional area.

**Figure (9):**
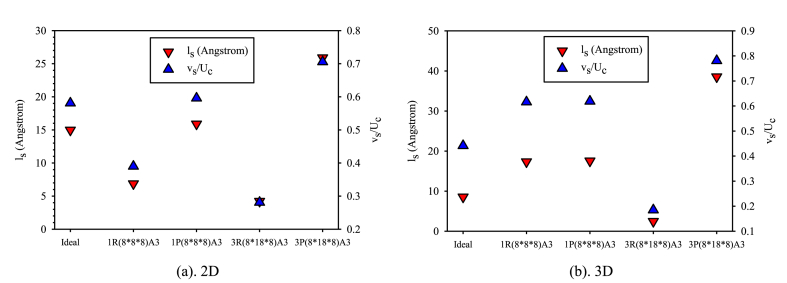
Changes in sliding length and sliding velocity ratio to central flow velocity withi a nanochannel encompassing various obstacle structures and dimensions comparing to a smooth state on the lower wall.

Fig. 9(a and b) presents diagrams illustrating the values of slip length and velocity ratio (the ratio of slip velocity on the lower wall to the central velocity of atoms) in both 2D and 3D nanochannels, considering variations in obstacle dimensions with values of 1 and 3. slip on the wall enhances the flow rate. Comparing the two and three-dimensional diagrams reveals that the slip length's behavior changes proportionally to the slip velocity ratio, and the patterns of change for these two parameters are similar. Based on the alignment of the sliding length diagrams and the velocity ratio, the values for these two parameters are higher in the three-dimensional state than in the two-dimensional state. This behavior can be attributed to the increased loss of velocity components for fluid atoms due to the presence of more solid atoms in the 3D nanochannels' walls. As the interactions between fluid-fluid and fluid-solid atoms intensify in the 3D state compared to the 2D state, the loss of velocity values and fluid velocity profiles influence the slip length and velocity ratio values. On the other hand, the loss of fluid velocity due to the R-arranged and P-arranged obstacles, impact the velocity profile behavior in areas between the surface and bulk, respectively. R-arranged obstacles lead to a significant reduction in atom velocity near the wall, decreasing both slip length and velocity. In contrast, P-arranged obstacles affect the velocity in the central regions of the nanochannel (the bulk flow region). This causes a loss of fluid atom velocity in the center of the nanochannel while enhancing the fluid atoms' velocity near the wall, ultimately leading to an increase in both slip length and velocity ratio. As the dimensions of the obstacles increase, the aforementioned behavior becomes more pronounced for the mentioned factors. In a 2D nanochannel, the maximum slip length and velocity ratio on the lower wall relates to P structured obstacles within the bulk flow region. The presence of obstacles with a P arrangement influences the velocity of argon fluid atoms in the bulk region and the wall force field, creating a localized constriction. These obstacles influence the argon atoms' velocity within the bulk area and the wall force field, creating a localized constriction. This increases the central flow velocity and shifts the velocity profile to the lower wall, as a result, the slip length increases. The 3P arrangement within the nanochannel, provides the optimal case for enhancing slip velocity and length. The presence of an R structure increases the solid-fluid atom interaction in the vicinity of the lower solid wall because of the loss of fluid atom velocity. As a result, argon atoms' velocity approach the wall velocity limiting the flow slip in this area. The minimum amount of flow slip on the solid surface occurs with a 3R arrangement. The presence of obstacle structures in a P configuration narrows the cross-section, increases the maximum flow velocity, and deflects the flow profile towards the lower wall. Additionally, fluid atoms lose momentum, colliding with obstacles in an R arrangement in the vicinity of the surface. In a 3D nanochannel, the maximum slip velocity and slip length are associated with a 3P structure, and as the effects of flow velocity loss intensify near the wall, the minimum value is related to obstacles with a larger 3R structure.

**Figure (10):**
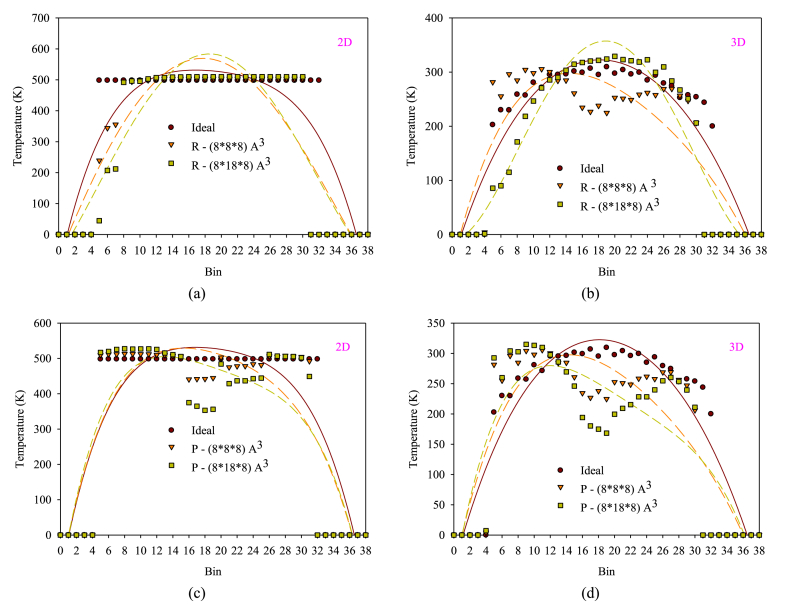
The diagrams of changes in the temperature of fluid atoms with changes in dimensions and different values of R and P.

Fig. 10(a–d) displays temperature change diagrams of fluid atoms with different dimensions and values of with R-arranged and P-arranged obstacles compared to smooth two- and three-dimensional nanochannels. Heat transfer in the profiles under study occurs because of the fluid-fluid and fluid-solid atoms temperature differences, with a significant portion of the heat transfer transferred between fluid atoms and the wall as a result of the collision and atomic diffusion mechanisms. Applying external force to fluid atoms increases their temperature, with the energy converted to the internal energy of atoms causing a temperature rise. An atom with a higher temperature has a higher kinetic energy, resulting in strong atomic collisions between fluid-fluid and fluid-solid atoms. This behavior leads to a variation in temperature distribution in different regions of the nanochannel. Any factor that disrupts this temperature distribution can create temperature gradients in the nanochannel. The presence of an R arrangement leads to some fluid flow atoms experiencing velocity loss in the sub-surface areas, resulting in energy loss through collisions with the wall at a lower temperature. This causes the temperature of these atoms to approach the wall temperature. A P arrangement in the nanochannel's central part can also intensify the effects of fluid-solid atom collisions, leading to a decrease in the temperature of fluid atoms in these areas. Increasing the dimensions of obstacles intensifies the aforementioned behaviors, resulting in a stronger diffusion phenomenon across other areas of the nanochannel. This causes temperature transfer from different fluid regions to areas further away from the solid surfaces, ultimately resulting in a more uniform temperature distribution. However, in the molecular free flow regime investigated in this study, the temperature distribution is not uniform with the wall temperature due to the violation of flow continuity accompanied by a temperature jump near the solid wall. The flow movement around the obstacles used results in significant changes in the velocity profile, with the indirect movement and change in the direction of fluid atoms leading to asymmetry in temperature profiles. As a result, the peak location of the temperature profile moves away from the center of the nanochannel with changes in the dimensions of the obstacles.

In all the investigated temperature graphs, the temperature depletion in the 3D nanochannel compared to the 2D nanochannel is caused by increased temperature exchange between fluid-liquid and solid-fluid atoms in the 3D state. The presence of added walls along the flow's height leads to more temperature exchange between the wall and the fluid. In the two-dimensional mode, the most significant amount of temperature reduction compared to the smooth nanochannel is related to the nanochannel with the presence of the barrier P structure with the dimensions of 8 × 18 × 8 Å^3^ and by 4.62 %. Also, the presence of roughness with the R structure with the same dimensions can create a higher heat exchange compared to the smaller P and R structures. In the 3D nanochannel, the presence of a P-arranged obstacle with dimensions of 8 × 18 × 8 Å^3^ has reduced the heat by 14.96 % in the nanochannel compared to the smooth state.

**Figure (11):**
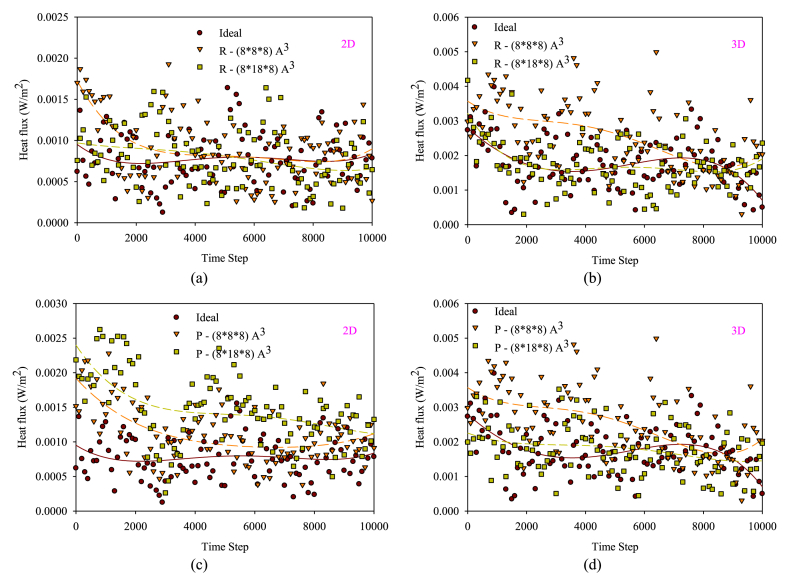
Percentage of changes in the heat flux of the flow field in the nanochannel with obstacles of various dimensions and arrangements comparing to the smooth state.

Fig. 11(a–d) displays the changes in the thermal flux of the flow field in the nanochannel with obstacles of varying dimensions compared to the smooth state. Thermal differences between atomic and fluid-wall atoms act as a stimulus for atom movement and heat flux distribution. In both two and three-dimensional states, there are significant changes in the amount of heat flux exchanged by fluid atoms. The heat flux distribution diagrams in the three-dimensional state are stronger due to the increased number of wall atoms, increased atomic collisions, and the gravitational force of the copper wall. As a result, the heat flux distribution in the three-dimensional state is more significant compared to the two-dimensional state. On the other hand, in a two-dimensional nanochannel, the temperature distribution in all areas of the nanochannel is primarily influenced by the collision mechanism in the bulk flow area, while molecular diffusion occurs in regions in the vicinity of the walls and rough surfaces. Therefore, the heat flux transfer in a two-dimensional nanochannel is more limited than in a three-dimensional state, as only one side of the nanochannel is in contact with the fluid. The obstacles can significantly affect the balance of temperature and heat flux distribution in various nanochannel areas, especially near the wall and the bulk flow section where the obstacles act as heat absorbers and by the collision with high energy atoms (fluid atoms affected by axial force), a notable amount of energy is absorbed. Consequent to the collision mechanism between fluid-solid atoms, more heat flux distribution occurs with the solid walls. The obstacles disrupt the kinetic balance of atoms and their distribution in the nanochannel, leading to a delayed temperature drop behavior due to thermal decay and interatomic equilibrium. This behavior ultimately leads to a more uniform temperature distribution and an increase in interatomic heat flux exchange, resulting in increased heat transfer to the wall.

**Figure (12):**
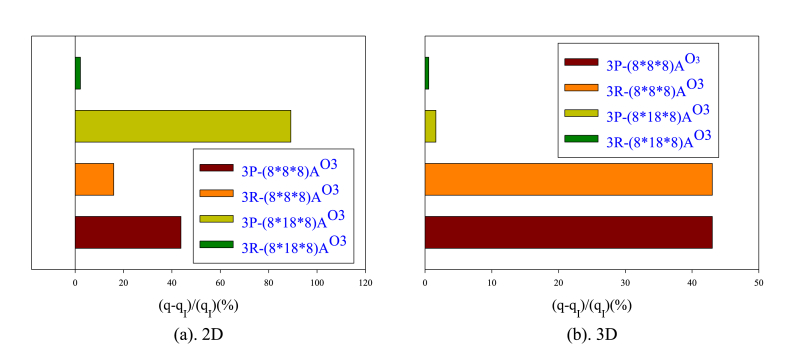
Bar charts of the percentage increase in heat flux in 2D and 3D nanochannels comparing to the smooth nanochannel with changing obstacle dimensions.

Fig. 12(a and b) displays bar charts of the percentage increase in heat flux in 2D and 3D nanochannels comparing to the smooth nanochannel with changing obstacle dimensions. More obstacles can accelerate heat transfer and improve heat flux distribution. In the two-dimensional state, the highest amount of heat exchange is related to the P-shaped obstacle structure and its dimension increase, while in the three-dimensional state, the obstacles with the R structure and their dimension strengthening lead to the highest heat exchange. The reason for this behavior lies in the improved heat transfer and the influence of the atomic collision mechanism in the central flow areas by the P-type arrangement. In the two-dimensional state, the fewer solid atoms is compensated by intensifying atomic collisions in the bulk flow area, leading to a maximum heat flux distribution compared to the R-arranged obstacels. In a three-dimensional nanochannel, enlarging the obstacles can deflect atomic collisions toward the wall. However, in areas close to the wall, increasing the dimensions of the obstacles will have less effect on interatomic heat exchange due to the presence of more solid atoms. It seems that the presence of smaller obstacles affects more areas above the wall, leading to a higher heat flux transfer.

**Figure (13):**
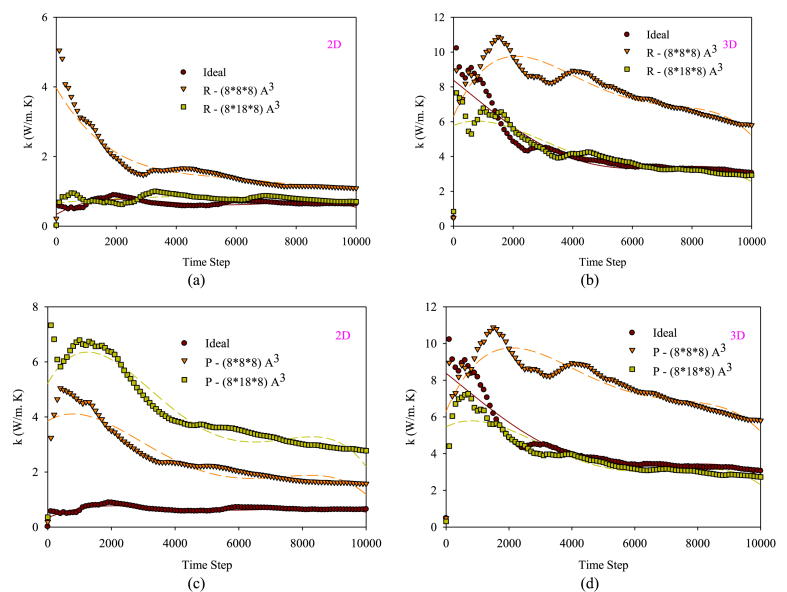
Time-dependent behavior of changes in fluid thermal conductivity in 2D and 3D nanochannels with one obstacle.

Fig. 13(a–d) shows the time-dependent behavior of changes in fluid thermal conductivity in 2D and 3D nanochannels for the obstacles with different arrangements and dimensional changes. In the thermal conductivity diagrams over time, the amount of thermal conductivity depends on changes in exchanged heat flux and temperature difference. Due to the increase in the collision mechanism, the heat flux exchanged between fluid atoms and the wall decreases in different directions in both 2D and 3D nanochannels.

In the early times of the system, after leaving equilibrium, because the temperature difference between the fluid and the wall is significant compared to each other, the consumption of atomic collisions due to losing heat exchange is not yet significant. However, over time, the temperature of the fluid and the wall get closer to each other, and heat exchange with the wall consumes a large part of the energy of fluid atoms. As the temperature of the fluid and the wall approaches each other, there is a decrease in the transfer of heat energy, resulting in a reduction in the internal energy, interatomic distance, and enthalpy of the argon atoms. This ultimately leads to a decrease in thermal conductivity. On the other hand, increasing the number of walls in the nanochannel enhances thermal conductivity significantly compared to the two-dimensional state, causing an increase in the level of thermal conductivity diagrams.

**Figure (14):**
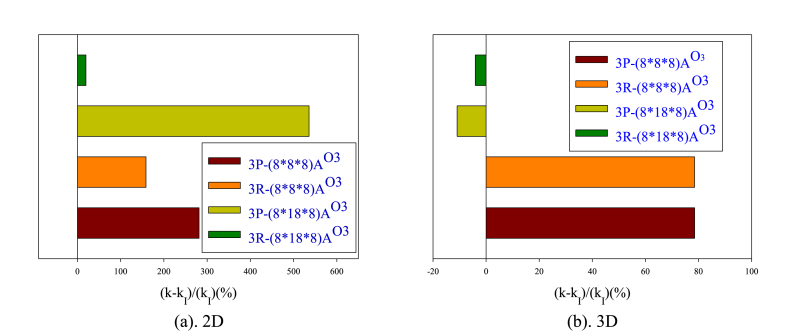
Percentage of changes in thermal conductivity of the flow field in nanochannels with different obstacle arrangements comparing to a smooth state.

Fig. 14(a and b) displays the percentage of changes in thermal conductivity of the flow field in 2D and 3D nanochannels with obstacles of varying dimensions compared to a smooth nanochannel. The obstacles in a 2D nanochannel increase the thermal conductivity. In order to enhance the thermal conductivity in a 2D nanochannel, P-type obstacles are more efficient than R-type. Similarly, in a 3D nanochannel, utilizing smaller obstacles is more advantageous as it leads to better atom distribution and increased interatomic collisions in the central region compared to the wall regions.

## 6 Conclusion

The article explores the MD of argon Poiseuille flow in a copper nanochannel, considering two and three dimensions while examining the impact of R and P-shaped obstacles. Using LAMMPS software, the study investigates changes in the dimensions of these factors and their effects on temperature and flow velocity distribution. The research aims to understand the behavior of surface and cross-sectional factors, particularly the R-arranged and P-arranged obstacles. For this purpose, the desired non-equilibrium solution to determine the flow behavior and heat transfer along with argon boiling with a time step of 1 fs and for 10,000 time steps in two and three-dimensional nanochannels has been studied. The research findings demonstrate that the fluid particles experience greater confrontation with the wall when they are close to it, and the wall force affects the distribution of fluid atoms. As the distance from the wall increases, the effect of the wall force on the distribution of fluid atoms in the nanochannel decreases, eventually reaching a minimum at the center of the nanochannel. In three-dimensional mode, there are more wall atoms than in two-dimensional mode. Therefore, the contrast between the fluid particles and the wall leads to more energy absorption by the wall from fluid atoms, resulting in less oscillation in the fluid density behavior. Near the wall, the enhancement of thermal conductivity is influenced by the collision mechanism between argon-copper atoms, while in areas above the surface and the center of the nanochannel, it is due to the diffusion, dispersion, and collision of argon-argon atoms. When larger obstacles are present, they cause a deviation of the velocity profile and the formation of maximum velocity. This behavior is particularly noticeable in the three-dimensional flow mode, where the flow profile's deviation towards the lower wall is more significant.

In the two-dimensional mode, the nanochannel with a P-shaped arrangement and dimensions of 8 × 18 × 8 Å^3^ exhibits the highest temperature reduction comparing to a smooth nanochannel, which is 4.62%. Additionally, the presence of larger R obstacles results in higher heat exchange compared to smaller obstacles. In a 3D nanochannel, the P-arranged obstacle structure with dimensions of 8 × 18 × 8 Å^3^ has caused a 14.96% reduction in heat transfer compared to the smooth state.

To continue this research, the authors recommend 1- investigation of the structure and flow parameters in non-square sections and the use of solid nanoparticles in argon fluid (nanofluid) and 2- studying of Couette Flow in rough nanochannel with square section.

## CRediT authorship contribution statement

**Omid Ali Akbari:** Software, Writing – original draft, Writing – review & editing. **Ebrahim Shirani:** Supervision, Writing – original draft, Writing – review & editing. **Mohsen Saghafian:** Investigation, Methodology, Writing – original draft, Writing – review & editing.

## Declaration of competing interest

The authors declare that they have no known competing financial interests or personal relationships that could have appeared to influence the work reported in this paper.

## Nomenclature

**Greek symbols**
Velocity, $\frac{Å}{\dot{\text{ps}}}$: v
Intermolecular distance, Å: σ
Intensity of interaction, eV: ε
Random number: ξ
Density, ${\text{gr}/Å}^{3}$: ρ
Mean free path ، nm: λ
Atomic mass, gr/mol: m
Degrees of freedom: d
Total internal energy, eV: $E_{total}$
Intermolecular force ، eV/Å: F
Time، ps: t
Boltzmann's constant, $8.6173324\times 10^{-5}\frac{eV}{K}$: $k_{B}$
Kinetic energy, eV: KE
Particle mass، gr: m
Number of particles: N
System pressure, bar: P
Potential energy، eV: PE
Location vector: r
distance between i and j atoms, Å: $r_{ij}$
Cut off radius, Å: $r_{c}$
Temperature, K: T
Actual temperature, K: $T_{A}$
Desired temperature, K: $T_{D}$
Hydraulic diameter, Å: $D_{H}$
